# Circulating microRNA sequencing revealed miRNome patterns in hematology and oncology patients aiding the prognosis of invasive aspergillosis

**DOI:** 10.1038/s41598-022-11239-z

**Published:** 2022-05-03

**Authors:** Gábor Fidler, Anna Anita Szilágyi-Rácz, Péter Dávid, Emese Tolnai, László Rejtő, Róbert Szász, Szilárd Póliska, Sándor Biró, Melinda Paholcsek

**Affiliations:** 1 Department of Human Genetics, Faculty of Medicine, University of Debrecen, Egyetem tér 1., 4032 Debrecen, Hungary; 2 Department of Hematology, Jósa András Teaching Hospital, Nyíregyháza, Hungary; 3 Division of Hematology, Institute of Internal Medicine, Faculty of Medicine, University of Debrecen, Debrecen, Hungary; 4 Department of Biochemistry and Molecular Biology, Faculty of Medicine, University of Debrecen, Debrecen, Hungary

**Keywords:** Molecular biology, Non-coding RNAs, miRNAs, Diagnostic markers

## Abstract

Invasive aspergillosis (IA) may occur as a serious complication of hematological malignancy. Delays in antifungal therapy can lead to an invasive disease resulting in high mortality. Currently, there are no well-established blood circulating microRNA biomarkers or laboratory tests which can be used to diagnose IA. Therefore, we aimed to define dysregulated miRNAs in hematology and oncology (HO) patients to identify biomarkers predisposing disease. We performed an in-depth analysis of high-throughput small transcriptome sequencing data obtained from the whole blood samples of our study cohort of 50 participants including 26 high-risk HO patients and 24 controls. By integrating in silico bioinformatic analyses of small noncoding RNA data, 57 miRNAs exhibiting significant expression differences (*P* < 0.05) were identified between IA-infected patients and non-IA HO patients. Among these, we found 36 differentially expressed miRNAs (DEMs) irrespective of HO malignancy. Of the top ranked DEMs, we found 14 significantly deregulated miRNAs, whose expression levels were successfully quantified by qRT-PCR. MiRNA target prediction revealed the involvement of IA related miRNAs in the biological pathways of tumorigenesis, the cell cycle, the immune response, cell differentiation and apoptosis.

## Introduction

Globally, the incidence of fungal infections is evidenced by the worrisome prevalence values of approximately 20 million cases of allergic fungal diseases and more than 1 million cases of invasive fungal infections (IFIs)^1,2^. IFIs are associated with dramatic mortality rates, ranging from 20 to 50% despite currently available powerful antifungal agents^3,4^.

Underscoring the burden of invasive aspergillosis (IA), a marked increase in disease prevalence was observed due to improved diagnostics, an overall escalation in the use of immunosuppressive therapies, and an increased number of organ transplantations performed in recent decades^5,6^. IA remains a major issue among patients who have undergone either stem cell or solid organ transplantation, with a prevalence of over 10%^7–10^. Considering the impact of the severity of infection, mold specific nucleic acid biomarkers and galactomannan antigen (GM) may prove to be valuable for a timely disease diagnosis.

Because of devastating statistics and high mortality rates, new and alternative diagnostic strategies are needed. To diagnose patients with IA in a timely manner, there is a comprehensive need to identify biomarkers with high specificity and sensitivity. Moreover, the application of minimally invasive procedures to obtain nucleic acid targets has become a research trend. Ultimately, biomarkers must be easily detectable with satisfactory positive and negative predictive values and must also discriminate hematology and oncology (HO) patients with or without IA.

MicroRNAs (miRNAs) are a class of typically small noncoding RNAs that can regulate gene expression posttranscriptionally through miRNA::mRNA interactions. By mediating the degradation of specific mRNAs, miRNAs reportedly play an important role in the pathogenesis of infectious diseases^11,12^. Because of their high diagnostic potential, stable, blood-born miRNAs have been evaluated as potential biomarkers of IFIs. Numerous studies have reported the aberrant expression of several miRNAs in various conditions, including hematological malignancies and bloodstream infections^13^. There is promising evidence that despite the lack of standardized protocols in disease prognosis and current clinical practice, miRNAs constitute a reliable tool for future use^14^.

In recent years, extraordinary progress has been made in terms of identifying miRNAs secreted in different body fluids. Cell-free miRNAs are not readily degraded by enzymes and are resistant to changes in temperature, storage, acids and alkalis that might also be exploited in IA^15^. In addition to the major technical difficulties of “liquid biopsy”, standardization is also needed for their successful clinical application^16^.

The evaluation of stable miRNA profiles in various biofluid samples is a feasible diagnostic procedure in clinical laboratories. Although previous studies revealed that differentially expressed miRNAs (DEMs) were associated with IFIs, currently, there are no validated prognostic miRNA markers associated with IA^17–19^.

Unlike SNPs and differential mRNA expressions, miRNAs are scarcely studied in fungal infections while having potential as a future host diagnostic and/or prognostic markers. This study provides a comprehensive dissection and discussion of differentially expressed miRNAs in hematology and oncology patients and thus presents a valuable resource on circulating biomarkers that might be involved in the progression of IA.

## Results

### Characteristics of the patient cohort

In this retrospective study, 50 participants (26 hematology and oncology patients and 24 healthy volunteers) were recruited from two hematology centers in Hungary (the University of Debrecen, Faculty of Medicine, Institute of Internal Medicine, Debrecen, Hungary and Institute of András Jósa County; and the Teaching Hospital, Division of Haematology, Nyíregyháza, Hungary) between May 2017 and November 2020. Participants in the cohort were balanced according to age (mean ± SD: 47.19 ± 13.93 years) but not sex (16 males/10 females). The vast majority of participants suffered from acute lymphoid leukemia (ALL, 53.85%), followed by acute myeloid leukemia (AML, 19.23%), non-Hodgkin lymphoma (NHL, 15.38%), myeloid sarcoma (MS, 7.69%), and chronic lymphocytic leukemia (CLL, 3.85%) (Table 1). 17 patients died during the study period. In case of 2 patients, IA was proven post-mortem by periodic acid-Schiff (PAS) staining. In total, 69.23% of the patients suffered from neutropenic fever, defined as a single oral temperature of ≥ 38.3 °C (101 °F) or a temperature of ≥ 38.0 °C (100.4 °F) sustained over a 1 h period, and 72.22% of these patients developed recurrent fever refractory to antibiotic treatment.

**Table 1:** Characteristics of the study participants.

Patients characteristics	Values
No. of patients	26
Male/female ratio	16:10
Median age (years) of males (range)	63 (33–71)
Median age (years) of females (range)	40 (25–52)
No. of ALL patients	14/26 (53.85%)
No. of AML patients	5/26 (19.23%)
No. of NHL patients	4/26 (15.38%)
No. of MS patients	2/26 (7.69%)
No. of CLL patients	1/26 (3.85%)
No. of P1 and P2 patients	7/26 (26.9%)
NF	18/26 (69.23%)
PMH	PAS+: 2/17; PAS−: 15/17

*ALL* acute lymphoid leukemia, *AML* acute myeloid leukemia, *NHL* non-Hodgkin lymphoma, *MS* myeloid sarcoma, *CLL* chronic lymphocytic leukemia, *P1* proven IA, *P2* probable IA, *NF* neutropenic fever, *PMH* postmortem histology, *PAS* periodic acid-Schiff.

### Sequencing the small RNA transcriptome of the patient cohort

The number of mapped cDNA reads was 3,450,028 ± 1,234,556 (75 bp each) per sample, totalling 81,075,658 reads per cDNA library. The majority of the sequences were 21–23 nucleotides long. More than 90% of clean reads were retained after filtering out low-quality tags, removing adaptors and cleaning up contaminants. Small RNA sequence types (represented by uniqueness) and length distribution were analysed. Overall, more than 95% (± 2%) of the clean reads were assigned as miRNAs.

### Quantitative analysis of the small noncoding RNA transcriptome revealed shared and unique miRNAs

**Figure 1:**
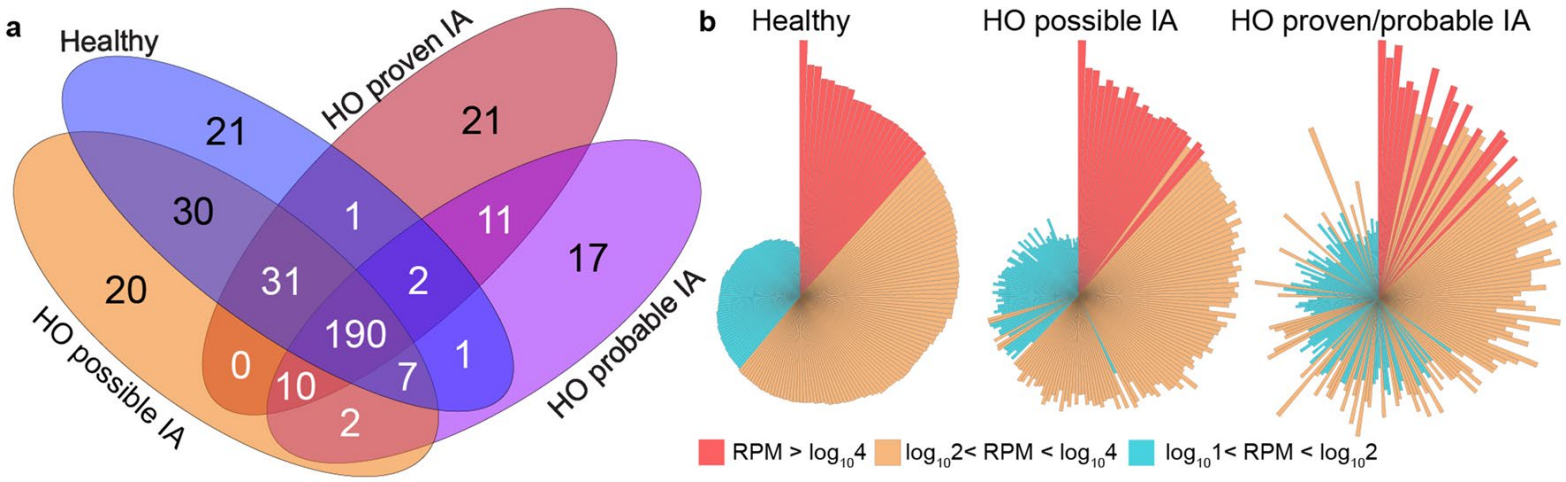
Conserved and unique miRNAs in different patient populations. (**a**) The number of individual and shared miRNAs was determined in healthy controls (H) and HO patients with proven (HO-proven), probable (HO-probable) and possible (HO-possible) IA. (**b**) Normalized distribution patterns of the 190 conserved miRNAs in the H, HO-possible and HO-proven/probable groups are shown as circo plots, where red, orange and blue correspond to miRNAs with high (RPM > log_10_4), medium (log_10_2 < RPM < log_10_4) and low (log_10_1 < RPM < log_10_2) read per million values, respectively. In every case the order of the miRNAs (the representative bars) was the same. Bar lengths refer to the log10 RPM sequence number.

In this study, high-throughput small RNA sequencing followed by in silico data analysis was used to detect unique and conserved circulating miRNAs in the study cohort, including healthy controls (n = 24) and HO patients with (HO-proven IA; n = 4, HO-probable IA; n = 3) or without (HO-possible IA; n = 19) IA. In total, 735 miRNAs were omitted from the analysis due to a very low read number (read per million [RPM < 10]) across all samples. We identified 364 miRNAs, with a read number above 10 (RPM > 10). We focused on these in our following analyses. Venn diagram was created to represent the number of miRNAs that were shared (“intersections”) and unique) between different datasets is (Fig. 1). Small RNA transcriptome compositions exhibited remarkable differences between our experimental groups (Fig. 1a). Overall, 190 miRNAs were uniformly present in all experimental groups, representing 19.02% of all identified miRNAs. By considering the global expression level distribution profiles of the common miRNAs, considerable differences were detected when comparing healthy controls to HO patients with or without IA (Fig. 1b). As shown, IA patient group exhibits remarkable expression changes in several miRNA read numbers. Analyses of the expressed conserved miRNAs revealed that most genes were uniformly up- or downregulated in the non-IA patient group. We also identified unique miRNAs in different experimental groups (Supplementary Fig. 1). In total, 21 and 20 miRNAs were present exclusively in healthy and non-aspergillosis HO controls. Based on our data we found 41 miRNAs that were presented in hemato-oncology patients with proven/probable IA. Of these, 21 were present in patients with proven IA (HO-proven), whereas 17 were present in patients with possible IA (HO-probable).

### DEMs in HO patients with IA

**Figure 2:**
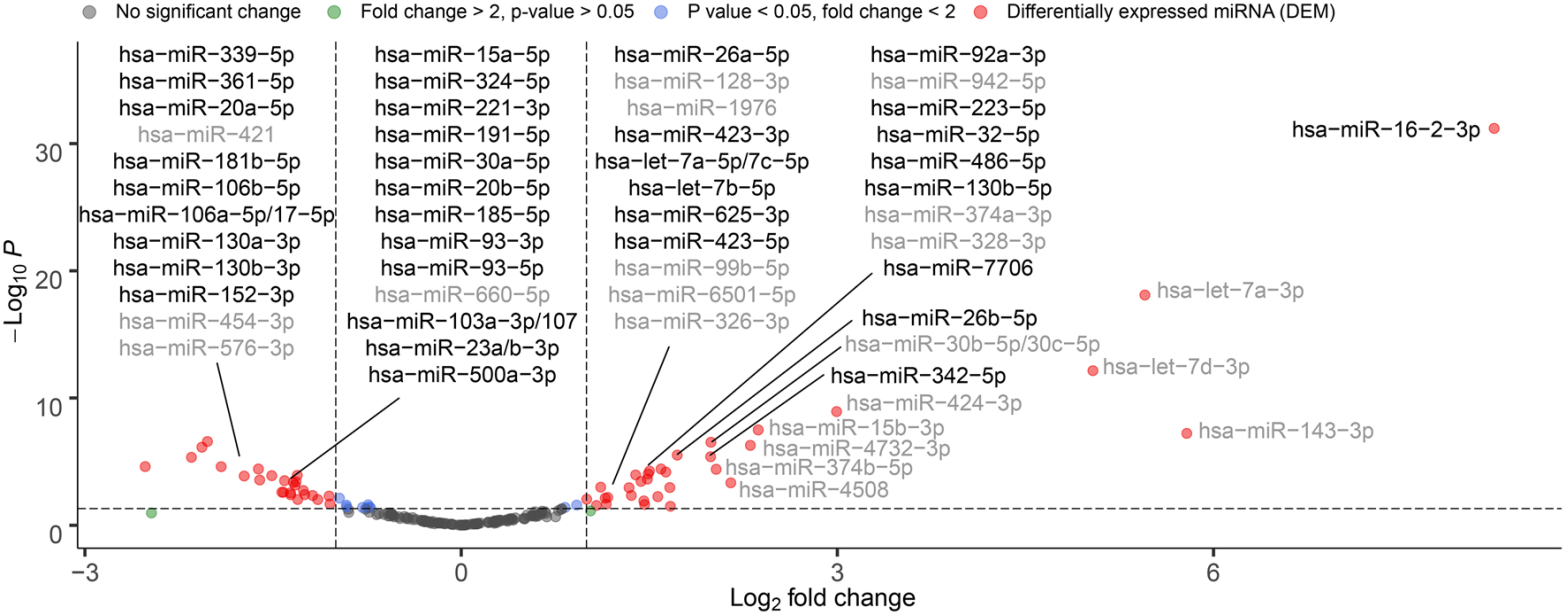
Results of differential expression analysis of the 190 conserved miRNAs by comparing HO patients with (HO-proven, HO-probable) or without (HO-possible) IA. Volcano plot represents the DEMs showing statistically significant overexpression and underexpression (according to the log_2_-transformed fold change in relation to the -log_10-_transformed P-value). The dashed line on the y-axis indicates the P-value = 0.05 threshold with statistically significant (*P* < 0.05) gene expression (up- and downregulation, respectively). Red circles indicate DEMs. Highlighted DEMs represent IA-specific miRNAs whose gene expression was not influenced by hematological malignancies.

Differential expression analysis was performed by retrieving the expressed reads of the 190 conserved miRNAs. Multiple miRNAs showed remarkable differences in expression when comparing HO patients with (HO-proven, HO-probable) or without (HO-possible) IA. Volcano plots were generated to identify the miRNAs showing fold differences with high statistical significance (P values ≤ 0.05) and expressing log_2_-fold changes greater than 1 and lower than − 1 (− 1 > fold change < 1) using the LIMMA statistical model (Fig. 2). Based on these criteria, which were considered stringent, we were able to reduce the number of conserved miRNAs to 57. Thereafter, we further identified 21 miRNAs in the IA group, whose miRNA expression profile was significantly different (twofold change with P < 0.05) in comparison to non-IA patients. Hereafter, we identified 36 IA-specific DEMs. Of these DEMs, the expression of 15 was upregulated, and the expression of 21 miRNAs was downregulated.

### Differential expression analysis of the circulating DEMs led to the identification of distinct clusters

**Figure 3:**
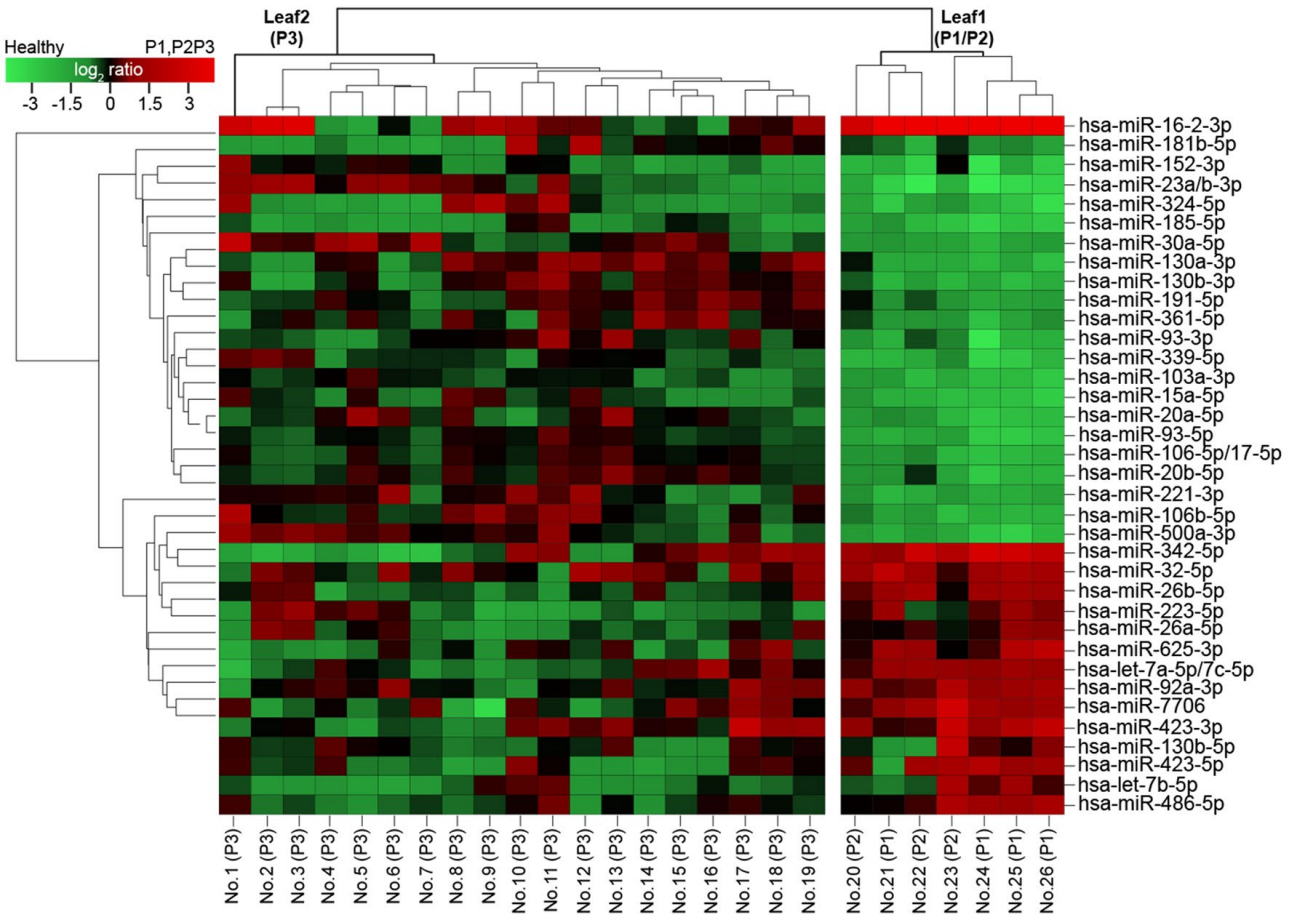
DEMs measured in the whole blood of HO patients diagnosed with or without IA. Hierarchical clustering was performed using the log_2_-transformed relative read counts of the 36 DEMs selected on the basis of the differential expression analysis in HO patients demonstrating a fold change difference greater than 2, *P* < 0.01 relative to healthy controls. Euclidian distances revealed two main clusters: Leaf 1 and Leaf 2. Leaf 1 represents patients with high-risk IA (HO-proven IA and HO-probable IA). Leaf 2 represents patients with low-risk IA (HO-possible IA).

The DEM patterns were also clustered to confirm the diagnostic potential of circulating miRNA signatures due to IA disease progression. A hierarchically clustered heatmap was constructed by relating the log_2_-fold change expression values of the 36 DEMs in patients with IA to those in healthy volunteers (Fig. 3). Of these miRNAs, 15 (hsa-miR-16-2-3p, hsa-miR-342-5p, hsa-miR-32-5p, hsa-miR-26b-5p, hsa-miR-223-5p, hsa-miR-26a-5p, hsa-miR-625-3p, hsa-let-7a-5p/7c-5p, hsa-miR-92a-3p, hsa-miR-7706, hsa-miR-423-3p, hsa-miR-130b-5p, hsa-miR-423-5p, hsa-let-7b-5p, hsa-miR-486-5p) were significantly upregulated while 21 (hsa-miR-181b-5p, hsa-miR-152-3p, hsa-miR-23a/b-3p, hsa-miR-324-5p, hsa-miR-185-5p, hsa-miR-30a-5p, hsa-miR-130a-3p, hsa-miR-130b-3p, hsa-miR-191-5p, hsa-miR-361-5p, hsa-miR-93-3p, hsa-miR-339-5p, hsa-miR-103a-3p, hsa-miR-15a-5p, hsa-miR-20a-5p, hsa-miR-93-5p, hsa-miR-106a-5p/17-5p, hsa-miR-20b-5p, hsa-miR-221-3p, hsa-miR-106b-5p, hsa-miR-500a-3p) were downregulated due to IA. Three miRNAs (hsa-miR-1976, hsa-miR-423-5p, hsa-let-7b-5p) exhibited inconsistent expression patterns in IA patients.

**Figure 4:**
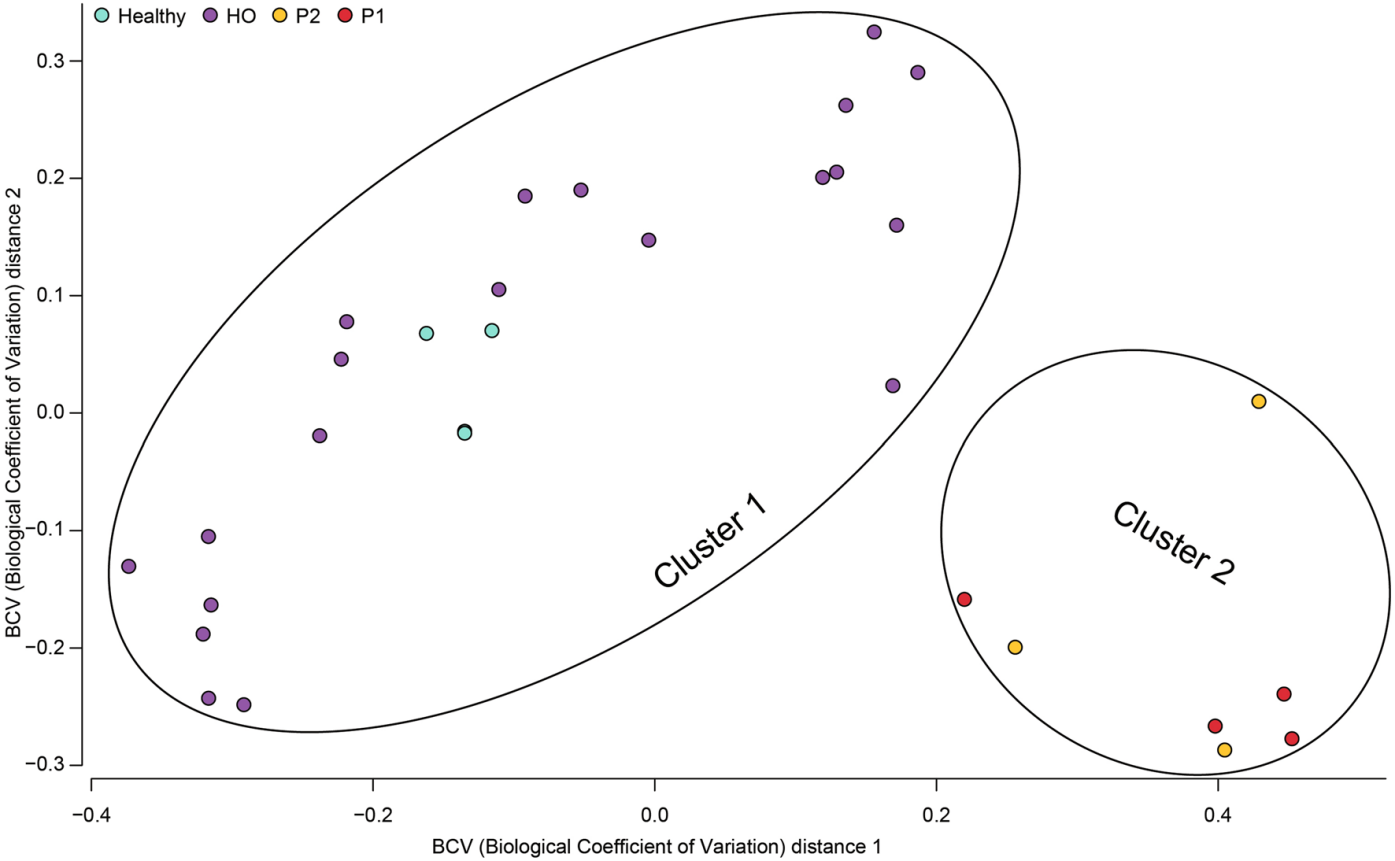
Multidimensional scaling (MDS) plot representing healthy controls (H) and HO patients diagnosed with (HO-proven, HO-probable) or without (HO-possible) IA. Red and orange circles represent proven and probable IA, gray circles represent healthy controls (H), and blue circles represent non-IA HO patients (possible IA). On the basis of distance matrices, the healthy controls and non-IA HO patients clustered together, and the IA-infected patients formed distinct clusters.

Beta diversity relationships are summarized in two-dimensional multi-dimensional scaling (MDS) scatterplots (Fig. 4). Each point represents a sample, and distances between points are representative of differences in DEM expression. Diversity plots were generated to represent the DEM-induced alterations discriminating IA patients from controls, resulting in nonoverlapping clusters (cluster 1 and cluster 2) and representing different spatial ordinations. The MDS plot shows that on the basis of the expression patterns of the IA-related miRNA signatures, it is possible to discriminate patients (HO-proven, and HO-probable IA) from noninfected (HO-possible IA and H, healthy) controls.

### Validation of the DEMs

**Figure 5:**
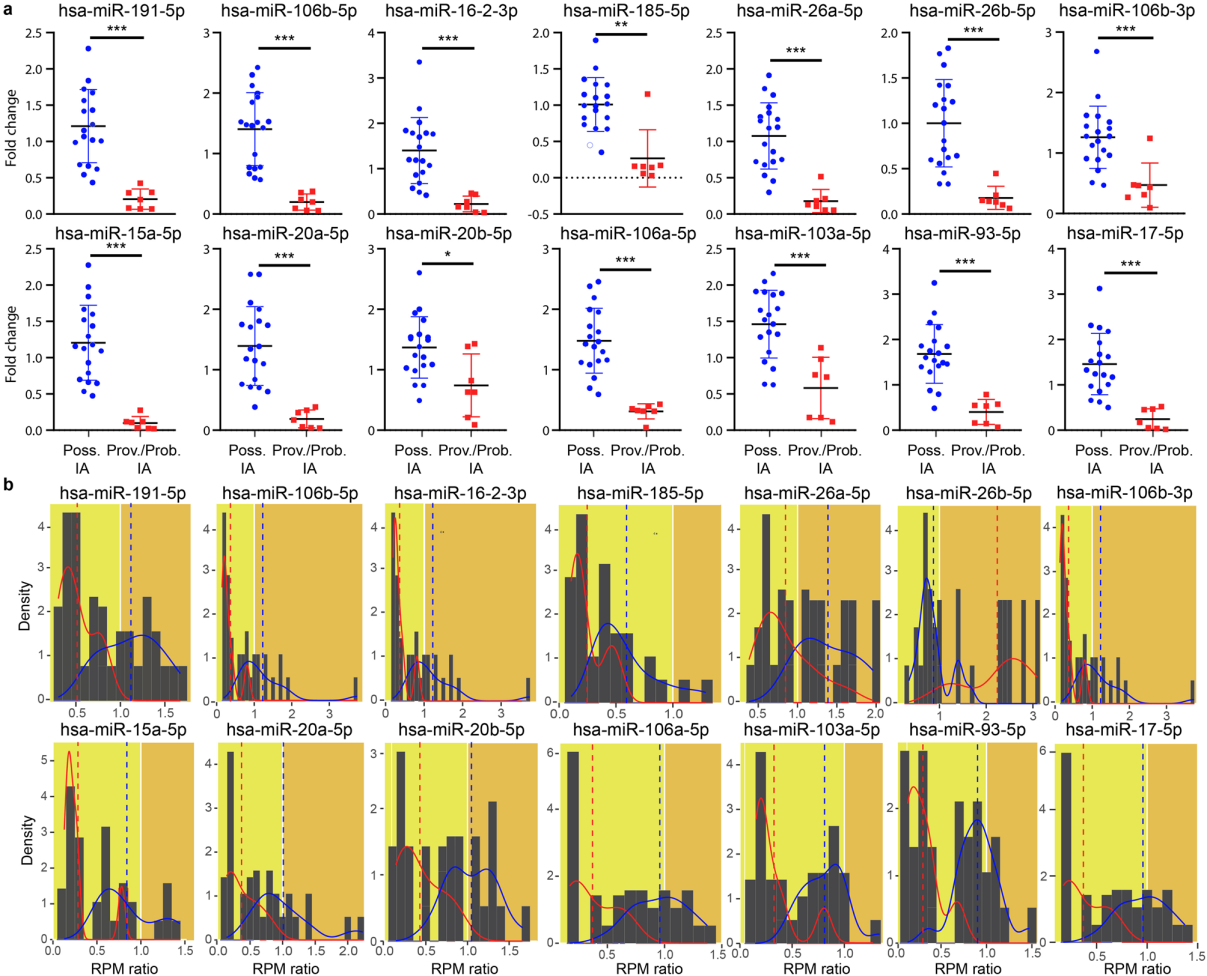
Representation of the fold changes in the qRT-PCR data and sequencing read numbers with their density distributions of validated DEMs measured in the whole blood of IA-infected and noninfected HO patients. (**a**) The downregulated gene expression of 14 DEMs was confirmed by qRT-PCR. Scatter plots represent the whole blood miRNA levels as relative miRNA concentrations with the formula 2-ΔCt (normalized to hsa-miR-181a-5p). Significant median differences in the miRNA levels between each group were determined by the nonparametric Mann–Whitney test (*P < 0.05, **P < 0.01, ***P < 0.001). (**b**) Density bars represent the normalized sequencing read numbers in patients relative to healthy controls, where the trend line indicates IA-infected (red) and noninfected (blue) patients.

An essential component of reliable quantitative reverse transcription PCR (qRT-PCR) analyses is the normalization of gene expression data because it controls for variations and allows comparisons of gene expression levels among different samples. An ideal reference gene must be stably expressed, abundant and without any significant variation in its expression status^20^. Due to high heterogeneity, there is no consensus for the best reference gene to be used to normalize miRNA gene expression data in HO patients. In this study, 20 candidate reference genes were investigated to normalize the RT-qPCR data, and their stability was evaluated. On the basis of the overall ranking data, hsa-miR-181a-5p was found to be the most stable, showing the highest stability among the 20 tested miRNAs (Supplementary Fig. 2). Of the 62 most abundant DEMs tested, 14 miRNAs were validated successfully by qRT-PCR across our sample groups. The 2-ΔΔCT method was used to quantify the relative fold changes in gene expression in patients (HO-proven and HO-probable vs. HO-possible) relative to healthy controls. To calculate relative changes in gene expression, for each sample, the normalized CT values of single miRNAs were related to the mean CT values measured in healthy controls according to Livak’s 2-ΔΔCT method (Fig. 5a). Based on these results, we found that the gene expression of 14 miRNAs (hsa-miR-191-5p, hsa-miR-106b-5p, hsa-miR-16-2-3p, hsa-miR-185-5p, hsa-miR-26a-5p, hsa-miR-26b-5p, hsa-miR-106b-3p, hsa-miR-15a-5p, hsa-miR-20a-5p, hsa-miR-20b-5p, hsa-miR-106a-5p, hsa-miR-103a-5p, hsa-miR-93-5p, hsa-miR-17-5p) exhibited significant changes due to IA.

To strengthen the congruent gene expression tendencies of small RNA-seq data and qRT-PCR measurements, the normalized read counts (in RPM) of IA patients relative to healthy controls with their density distributions were also determined throughout the IA-infected (HO-proven and HO-probable IA) vs. noninfected (HO-possible IA) hematology and oncology patients (Fig. 5b).

### Diagnostic performance of miRNA biomarkers from whole blood

**Figure 6:**
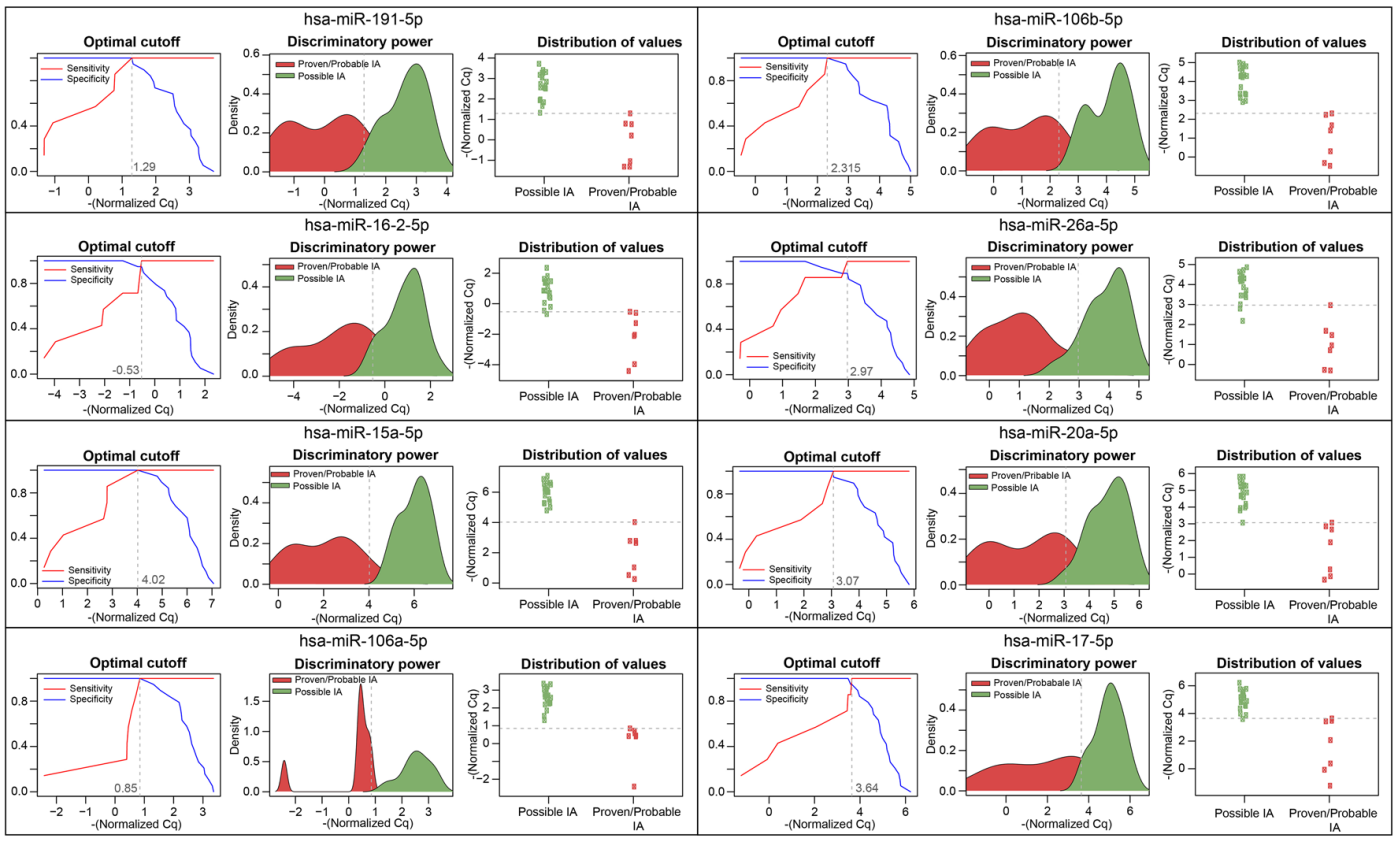
ROC curves were constructed to assess and visualize the performance of 8 preselected miRNAs. To measure the diagnostic accuracy of the miRNAs, relative fold changes were converted to qualitative (proven IA, probable IA vs. possible IA) indexes. The normalized CT values of eight miRNAs revealed their significant downregulation in IA-infected hematology and oncology patients (proven/probable) relative to noninfected patients (possible), indicating IA. Line graphs were used to calculate the optimal cutoff points. Scatter graphs represent the distribution of the CT values in cases and controls, and area plots represent the discriminatory power of the biomarkers.

To estimate the capabilities of DEMs to discriminate aspergillosis-infected and noninfected patients from whole blood samples, receiver operating characteristic (ROC) curve analyses were applied (Fig. 6). On the basis of qRT-PCR-validated gene expression analyses, eight DEMs were found to display high discriminatory power (hsa-miR-191-5p, hsa-miR-106b-5p, hsa-miR-16-2-3p, hsa-miR-26a-5p, hsa-miR-15a-5p, hsa-miR-20a-5p, hsa-miR-106a-5p and hsa-miR-17-5p). All of these miRNAs were downregulated in the IA confirmed group, representing statistically significant fold changes (*P* < 0.05) relative to noninfected controls. Five miRNAs (hsa-miR-191-5p, hsa-miR-106b-5p, hsa-miR-15a-5p, hsa-miR-20a-5p, hsa-miR-106a-5p) demonstrated excellent discriminatory power, with AUC values of 1. Three additional miRNAs (hsa-miR-16-2-3p, hsa-miR-26a-5p and hsa-miR-17-5p) displayed AUC values greater than 98%. In addition to examining the distribution of the CT values and the discriminatory power of the miRNAs, normalized CT values for cases (proven and probable IA) and controls (possible IA) were also dichotomized by mapping the sensitivity values in relation to 1-specificity to estimate the optimal cutoff values for these biomarkers. In every case, we also estimated the optimal cutoffs, defined as the points that maximized sensitivity and specificity.

### Computational prediction reveals genes and biological functions affected by dysregulated miRNAs

The biological effects of miRNAs depend on various factors. Predicted interactions were retrieved from the integrated databases. Target recognition refers to the process by which mature miRNAs recognize their complementary mRNA sequences and regulate gene expression. An online webtool algorithm, miRabel, was employed to predict the target genes or biological pathways related to the dysregulated miRNAs considering their evolutionary conservation, Watson–Crick complementarity, and thermodynamic properties between the seed region of the miRNA and its target mRNA^21^.

**Figure 7:**
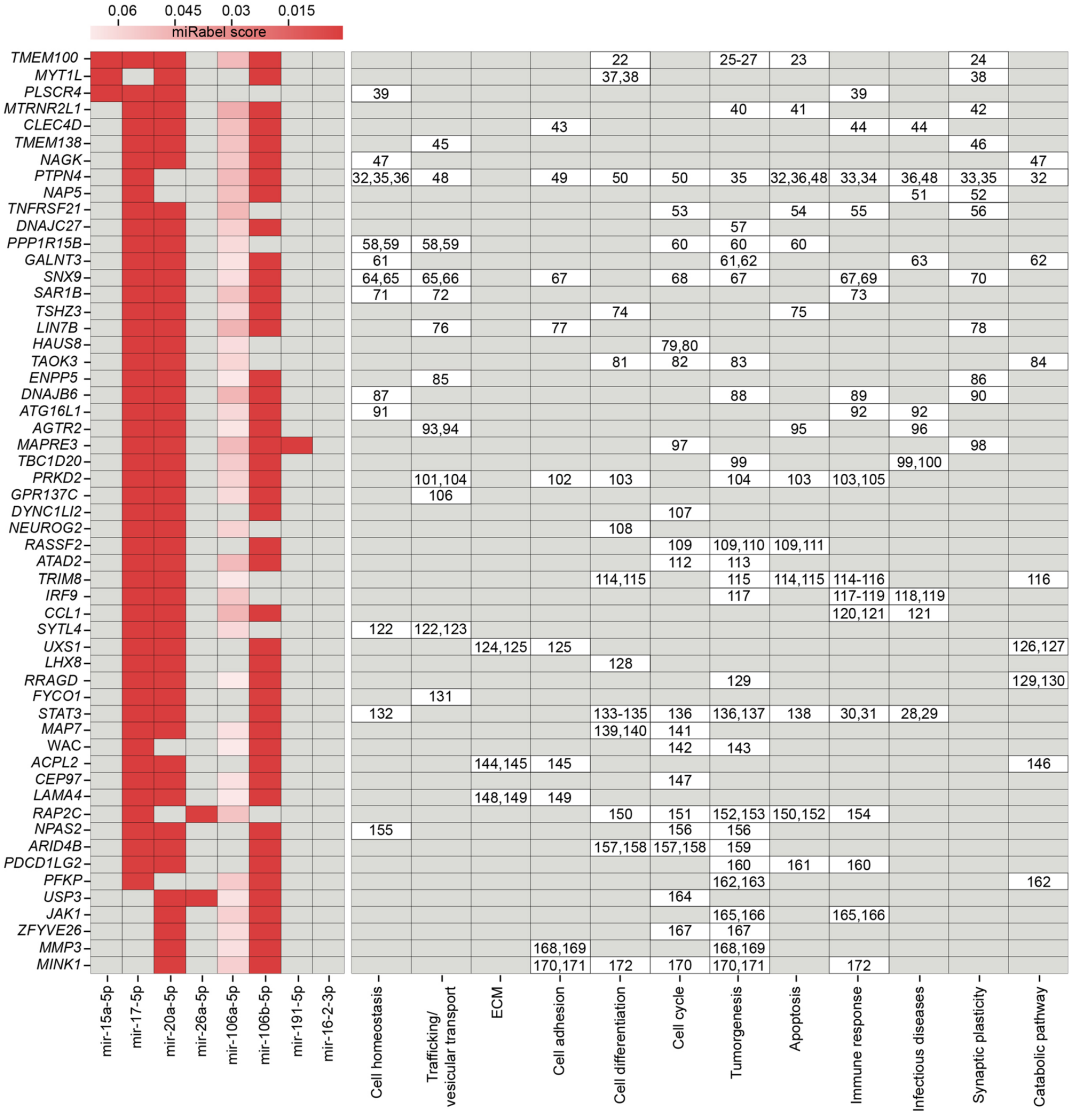
Representation of the target genes affected by dysregulated miRNAs and the metabolic pathways involved. miRabel scores were generated to rank miRNA::mRNA interactions and are inversely proportional to the rank of a given interaction. As suggested by the developers, the threshold was set to 0.05. The heatmap represents the miRabel scores. Numbers in the right part of the figure point to references supporting the association of the miRNAs, corresponding genes and pathways.

On the basis of in silico data predictions, we generated a list of 55 target genes whose expression might be posttranscriptionally influenced by at least three IA-specific DEMs (Fig. 7).

Pathway analysis was performed with the KEGG database (Supplementary Fig. 3). On the basis of the in silico pathway analyses, twelve relevant biological functions, “cell homeostasis”, “trafficking/vascular transport”, “extracellular matrix (ECM)”, “cell adhesion”, “cell differentiation”, “cell cycle”, “tumorigenesis”, “apoptosis”, “immune response”, “infectious diseases”, “synaptic plasticity” and “catabolic pathways”, were found to be influenced by changes in the IA-affected miRNAs. Of these, tumorigenesis (27 hits), the cell cycle (20 hits), the immune response (17 hits), cell differentiation (14 hits) and apoptosis (13 hits) were the top 5 affected pathways. The associations of these miRNAs with the regulated genes of these pathways were experimentally proven by other previous studies^22–172^.

## Discussion

IFIs are a major cause of mortality in immunosuppressed patients. IA is the most common mold infection in immunocompromised hosts associated with a poor prognosis and high mortality if diagnosis is delayed. Missed diagnoses are encountered when appropriate diagnostic tools are not available, especially in low-income and middle-income areas^173^. Currently, the early detection of IA is very difficult because most patients have nonspecific symptoms, leading to postponement of a correct diagnosis and therapy. The identification of easily accessible, noninvasive, blood-born biomarkers at early stages of disease progression is crucial for the evaluation of high-risk subjects to establish follow-up strategies.

Technological advances in high-throughput molecular methods have provided possibilities to detect miRNA expression patterns in different biological samples. Obtaining circulating miRNAs from the blood represents a minimally invasive method for the early detection of disease or to aid in treatment options. The discovery of disease-specific miRNA expression signatures is essential to obtain an accurate diagnosis and to better understand disease pathology. Blood is an easily obtained biofluid that can be used to identify biomarkers^174^.

Considering the increasing evidence from the literature showing that the dysregulated expression of miRNAs plays a pivotal role in various infections, we proposed that certain circulating miRNAs may play a significant role in the outcome of IA, suggesting that their relative gene expression levels might also serve as indicators of disease progression^175,176^.

By performing small RNA sequencing, this study has undertaken a comprehensive exploratory evaluation to establish the full repertoire of circulating miRNAs in whole blood among critically ill patients at high risk of IFIs. Circulating miRNAs were also recently recognized as promising disease biomarkers in infectious diseases, but relatively few studies have examined their role in IA. The regulatory roles of hsa-miR-132-5p and hsa-miR-212-5p have been associated with fungal infections^18^.

By considering baseline patient characteristics and underlying malignancies, our primary goal was to decipher aberrant miRNA expression patterns. We hypothesized that by comparing distinct miRNA-seq profiles of shared miRNAs between cases and controls, we can decipher specific prognostic markers that can aid in disease diagnosis. In this study, the most abundant, conserved miRNAs constituted 19.02% of the pool.

Differential expression analysis was employed to systematically search the small RNA transcriptome data for a subset of circulating miRNAs representing the most promising combinations of DEMs. Of the potential DEMs, we identified a subset of miRNAs whose expression signatures are unlikely influenced by hematological malignancy but likely to indicators of IA infection. In miRNA-based biofluid analyses, when a continuous variable is considered a diagnostic marker, the method adopted for data normalization and the choice of the reference gene is very important. Using hsa-miR-181a-5p as a reference, we found that dysregulated hsa-miR-191-5p, hsa-miR-106b-5p, hsa-miR-16-2-3p, hsa-miR-26a-5p, hsa-miR-15a-5p, hsa-miR-20a-5p, hsa-miR-106a-5p and hsa-miR-17-5p showed strong discriminatory power, with AUC values greater than 98%.

Despite continued progress, target prediction of miRNAs remains a challenge, since aggregated databases often show inconsistent results. To date, approximately 3000 mature human miRNAs have been referenced in miRBase, but several recent studies suggest that there may be a larger number^177^. Furthermore, the bioinformatics identification of miRNA targets remains a challenge because mammalian miRNAs are characterized by poor homology toward their target sequence^21^. Confirmation of the potential biological relevance of these predicted targets is laborious, and it was not the goal of the current project. In relation to IA, the in silico analysis of miRNA-influenced genes suggested an enrichment of pathways associated with tumorigenesis, the cell cycle, the immune response, cell differentation and apoptosis.

Interestingly, hsa-miR-16-2-3p was shown to have no influence on these genes, and hsa-miR-191-5p affected only the gene encoding the product of the microtubule-associated protein RP/EB family member 3 (*MAPRE3*). As a member of the transmembrane protein family, the product of the gene transmembrane protein 100 (*TMEM100*) was also experimentally proven to be involved in cell differentiation, apoptosis and synaptic plasticity^22–24^. Two genes, *TMEM100* and *MAPRE3*, were epigenetically influenced by five miRNAs, and both were markedly targeted by hsa-miR-17-5p (*TMEM100* miRabel score: 0.00056, *MAPRE3* miRabel score: 0.00069), hsa-miR-20a-5p (*TMEM100* miRabel score: 0.00048, *MAPRE3* miRabel score: 0.0012), and hsa-miR-106b-5p (*TMEM100* miRabel score: 0.00036, *MAPRE3* miRabel score: 0.00108) and weakly targeted by hsa-miR-106a-5p (*TMEM100* miRabel score: 0.0485, *MAPRE3* miRabel score: 0.0488). Previous studies have also implied a direct link between *TMEM100* and miR-106b-5p related to tumorigenesis^25–27^.

Based on our data, dysregulated hsa-miR-17-5p, hsa-miR-20a-5p and hsa-miR-106b-5p target the signal transducer and activator of transcription 3 (*STAT3*) gene in HO-IA patients. The *STAT3* gene encoding the transcription factor, which is a member of the STAT protein has also been proven to play an important regulatory role in both bacterial and fungal infectious diseases^28,29^. A defect in the IFN-γ response in *STAT3*-deficient patients has already been proven upon stimulation with heat-killed *Staphylococcus aureus* and *Candida albicans*^30,31^.

In addition, the involvement of the tyrosine protein phosphatase nonreceptor type 4 protein, encoded by the *PTPN4* gene, in infectious diseases was also proven that also plays a role in immunity and cell homeostasis^32–36^.

We found that the *PTPN4*, *STAT3* and *RAP2C* genes were the main targets with important roles in relevant biological processes. In humans, loss-of-function mutations of the *STAT3* gene are frequently associated with susceptibility to bacterial as well as fungal infections^178^. Francois Danion and colleagues proved that *STAT3*-deficient patients with aspergillosis were associated with a defective adaptive immune response against *A. fumigatus* infection and produced lower levels of cytokines, including IFN-γ, IL-17, and IL-22^178^. Based on their estimations, one major protective host mechanism against *A. fumigatus* infection is via IFN-γ. Furthermore, a recent study showed that the majority of lung-derived T cells upon *A. fumigatus* infection were Th17 cells, suggesting that the decreased production of Th1 and Th17 cytokines in *STAT3*-deficient patients could be the reason for their susceptibility to *A. fumigatus*^179,180^.

The tumor suppressor protein encoding *TMEM100* gene was found to be targeted by five IA-related miRNA biomarkers; hsa-miR-15a-5p, hsa-miR-17-5p, hsa-miR-20a-5p and hsa-miR-106a/b-5p. The fact that all of the miRNAs targeting *TMEM100* have shown significant changes in gene expression in HO patients with aspergillosis also suggests its involvement in both potentially oncogenic and infection-related biological pathways^26^.

Interestingly, in previous studies, the regulatory roles of some of these miRNAs were associated with infectious mycobacterial disorders. By binding to the 3’-untranslated region of cathepsin S (CtsS) mRNA, hsa-miR-106b-5p was found to be involved in the posttranscriptional gene regulation of CtsS during mycobacterial infection^181^. Additionally, the involvement of miR-26a-5p was defined upon *Mycobacterium tuberculosis* infection by targeting the IFNγ signaling cascade^182,183^. Finally, by targeting *STAT3*, the involvement of hsa-miR-17-5p in the regulation of tuberculosis-induced autophagy in macrophages was also proven^184^.

The experimental design of this study led us to decipher complex miRNA signatures associated with IA by integrating small RNA sequencing and multiple bioinformatics tools. A miRNA::mRNA regulatory network was also constructed to investigate relevant downstream molecular mechanisms of the predicted targeted genes of the captured miRNAs. To our knowledge, this is the first effort to understand the levels of blood-born, circulating miRNAs per IA to identify stable, abundant disease-specific biomarkers.

Our results suggest that some DEMs have the potential to serve as good and abundant blood-born biomarkers for IA. Our data may also lead to a better understanding disease pathogenesis and provide insight into the complexity and diversity of small RNA molecules that regulate immunodeficient IA.

## Study limitations

Regarding its incidence, IA can be considered a rare disorder. Based on epidemiological data on IA the estimated occurrence of IA is 5–13% in HSCT recipients and 10–20% in patients receiving intensive chemotherapy for leukemia^185–187^. In our study, disease prevalence exceeded 25% which might be explained by the relatively small hemato-oncology population size (HO-proven/probable IA). Due to the imbalance and limited size of the study cohort, this study may be considered exploratory.

For a higher level of confidence, differential expression of the miRNome should be studied in an extended cohort by recruiting patients from a more diverse HO population. Therefore, validation of the results in an extended population with a broader range of patients is needed.

There is a lack of standardized protocols for miRNA extraction or quality and quantity assessment either. Furthermore, due to the high levels of endogenous ribonuclease activity and low RNA content quantity of circulating miRNAs seem to vary widely between commercially available kits^188^. Because of the poor RNA yield many profiling methods are using total RNA. Furthermore, nanospectrophotometry is highly sensitive for low RNA concentration, resulting to poor quality criteria.

It also needs to be considered, that many miRNAs reported as circulating cancer biomarkers reflect a secondary effect on blood cells rather than a tumor cell-specific origin^189^. The fact, that circulating miRNAs are influenced by blood cell counts and hemolysis, establishing a correct and optimal miRNA extraction is crucial for biomarker studies. While of major interest for future biomarker development, this study presents a retrospective evaluation of our patient cohort, and no prospective validation of the identified miRNAs in independent cohorts has been performed. Therefore, for future studies of circulating miRNA biomarkers that are expressed in blood cells, miRNA expression levels should also be interpreted in light of blood cell counts.

## Conclusions

The most recent advances in the diagnosis of invasive fungal diseases indicate miRNAs. However, the number of patients at risk of IA is increasing globally, and data on disease-specific circulating miRNAs are scant. Microbiological laboratories still struggle to achieve a timely and adequate diagnosis. Numerous scientists tend to identify biomarkers that could help in the early diagnosis of IA. Therefore, the discovery of specific predisposing factors is essential to obtain an accurate diagnosis and a better understanding of disease pathophysiology. As circulating miRNAs are promising biomarkers for various diseases, in this study, we analyzed the small RNA transcriptomes of HO patients and healthy controls through next-generation sequencing to reveal IA-specific miRNA expression patterns. The identification of IA-specific miRNA signatures might also be essential for the elucidation of disease pathophysiology.

## Materials and methods

### Patient population

This retrospective case–control study was performed from May 2017 to November 2020 and involved two hematology centers in Hungary: the University of Debrecen, Faculty of Medicine, Institute of Internal Medicine, Debrecen, Hungary; and the Institute of András Jósa County and Teaching Hospital, Division of Hematology, Nyíregyháza, Hungary. The patient population comprised 26 adults: 16 males, with a median age of 63 (range 33–71) years, and 10 females, with a median age of 40 (range 25–52) years, with different hematological malignancies (mainly acute leukemia: 73.08%) receiving stem cell transplantation and intensive chemotherapy (neutrophil count < 0.5 × 109 cells/L) (Table 1). Patients who developed neutropenic fever (NF) (temperature > 38 °C of fever recorded twice or > 38.5 °C recorded once) were recruited. Children aged < 17 years were excluded from the study. Twenty-four healthy controls with no previous history of hematological and oncological diseases were also included [median age: 36 years (range 25–52)].

### Stratification of episodes

Patients were retrospectively stratified as follows using standard criteria according to the revised European Organization for the Research and Treatment of Cancer/Mycosis Study Group (EORTC/MSG)^190^: proven IA—4 patients (15.38%), probable IA—3 patients (11.54%), and possible IA—19 patients (73.08%).

### RNA extraction, quantification and quality control

Whole blood was drawn from patients and collected into EDTA-coated tubes for microRNA analyses. Analyses were carried out in a class II laminar-flow cabinet to avoid environmental contamination. Total RNA was extracted using a miRNeasy Serum/Plasma Kit (Qiagen, Hilden, Germany). RNA extraction was performed on 250 μl of whole blood according to the manufacturer’s instructions. A no template control (NTC) of nuclease-free water was purified with the samples. RNA quantity was measured in each sample using fluorometric quantification (Qubit™ 4 Fluorometer, Thermo Fisher Scientific, USA) with a Qubit miRNA Assay Kit (Q32881, Invitrogen by Thermo Fisher Scientific, USA). The RNA integrity number (RIN) and RNA quality were measured using two different methods: spectrophotometry (NanoDrop™ 2000 Spectrophotometer, Thermo Scientific) and automated electrophoresis with Agilent 4200 Tapestation System (G2991A, Agilent Technologies, USA) using RNA ScreenTape (5067–5576, Agilent Technologies, USA) and RNA ScreenTape Buffer (5067–5577, Agilent Technologies, USA). For all samples, the RIN value was above 5. After RNA quality control, the purified RNA samples were stored at − 80 °C.

### Library preparation and sequencing

Libraries for small RNA sequencing were prepared using a NEBNext® Small RNA Library Prep Set for Illumina^®^ (New England Biolabs Inc., United Kingdom) following the manufacturer’s instructions. Two sequencing runs were performed, samples were divided to batches in a random manner and both runs contained samples from all study groups in order to address batch effects (Supplementary Fig. 4). Six microliters of 500 ng total RNA was used as the starting material to prepare the libraries. Multiplex adapter ligations (using 3′ and 5′ SR adaptors), reverse transcription primer hybridization, reverse transcription reactions and PCR amplifications were performed as described in the protocol. After PCR amplification, the cDNA constructs were purified with a QIAQuick PCR Purification Kit (28104, Qiagen, Hilden, Germany) and MagSI-NGS^PREP^ Plus beads (MDKT00010075, magtivio BV, The Netherlands) following the modifications suggested in the NEBNext Multiplex Small RNA Library Prep protocol. Size selection of the amplified cDNA constructs was performed using E-Gel® EX 2% Agarose (G401002, Invitrogen by Thermo Fisher Scientific, Israel) with an E-Gel™ Power Snap Electrophoresis Device (G8100, Invitrogen by Thermo Fisher Scientific, Singapore) following the manufacturer’s protocol. The 150 nt bands correspond to adapter-ligated constructs derived from RNA fragments of 21 to 30 nt in length. An agarose slice was excised from the gel, melted, and purified using a QIAQuick Gel Extraction Kit (28,704, Qiagen, Hilden, Germany) following the manufacturer’s recommended protocol. The purified cDNA libraries were checked on Agilent 4200 Tapestation System using D1000 ScreenTape (5067–5582, Agilent Technologies, USA) and D1000 Sample Buffer (5067–5602, Agilent Technologies, USA). All libraries were adjusted to a concentration of 4 nM using 10 mM Tris (pH 8.5) as the diluent and pooled in the same proportion. Thereafter, libraries were denatured with 0.2 N NaOH. A standard 1% PhiX Control Library (Illumina, USA) was also denatured and used as an internal control. Finally, the libraries and PhiX control were sequenced on an Illumina NextSeq 550 Sequencing System (Illumina, USA) with read lengths of 75 base pairs and 3.5 million single-end reads per sample, on average.

### Bioinformatic and statistical analyses

#### Sample preprocessing and determining DEMs

The demultiplexed library was checked for residual adapter sequences with Cutadapt software, and AGATCGGAAGAGCACACGTCTGAACTCCAGTCAC query sequences were filtered^191^. Read qualities were assessed using the FastQC program. We summarized the sequencing quality across samples grouped by batches in order to detect outliers with poor quality (Supplementary Fig. 4). Additional trimming was performed with Trimmomatic (4:20 sliding window parameter)^192^. miRNA annotation was performed with miRge 2.0 software^193^. Sequencing reads were divided into two partitions with a target read length threshold of 28 bases. For the lower portion (< 28 bases) annotation reports showed that circa 95% of the reads were assigned miRNAs, while for the upper part of the reads (> 28 bases) no miRNAs were detected. Differential expression analysis was performed with the edgeR R package. Libraries in the program were normalized by trimmed mean of M values (TMM). Volcano plots from the edgeR result were generated using the EnhancedVolcano R package^194^. Statistical comparisons among groups were also checked with nonparametric Kruskal–Wallis test where sequencing read numbers were converted to RPM (reads per million reads) in order to normalize libraries. P values were adjusted with Benjamini–Hochberg method, and P < 0.05 was determined as significant difference. Additionally, clustermap was generated in Python (ver3.6.14) with the seaborn package (0.11.1)^195^, where dendograms were also created with hierarchical agglomerative clustering.

#### Diagnostic performances of the DEMs

The diagnostic values of the preselected miRNA biomarkers were measured by easyROC, a web-based tool for ROC curve analysis^196^. The ROC curve was edited by plotting the true positive rates (sensitivity values on the y-axis) versus the false positive rates (1-specificity values on the x-axis). The area under the ROC curve (AUC) was also calculated and used as an accuracy index to evaluate the diagnostic performances of the selected miRNAs.

### Target and pathway prediction

miRabel^21^, a miRNA target prediction tool, was used to determine the gene targets of the 7 selected miRNAs. For every miRNA, the top 100 hits were chosen according to the generated miRabel scores. Pathway analysis was carried out with the Kyoto Encyclopedia of Genes and Genomes (KEGG) database^197–199^.

### Validation of miR-seq data by qRT-PCR

Total RNA (1.5 ng) was used for miRNA-specific reverse transcription using a TaqMan™ Advanced miRNA cDNA Synthesis Kit (Thermo Fisher Scientific, USA). Quantitative real-time PCR with 62 TaqMan™ Gene Expression Assays (Thermo Fisher Scientific, USA) was performed to detect miRNA expression profiles in 3 independent technical repeats, including negative controls (no template from RNA isolation and reverse transcription), using a LightCycler^®^ 480 Real-Time PCR System (Roche Diagnostics, Risch-Rotkreuz Switzerland). PCR conditions were as follows: 20 s at 95 °C, 50 cycles of 3 s at 95 °C and 30 s at 60 °C followed by 1 cycle of 3 min at 37 °C. To identify a stable endogenous miRNA control in whole blood samples from healthy controls and study participants, twenty candidate miRNAs were selected by RefFinder^200^. Among the 20 reference miRNAs, hsa-miR-181a-5p was the most stable and used for normalization.

### Postmortem histology

The specificity of Aspergillus infection morphology via PAS staining was addressed because open lung biopsy was performed via postmortem thoracotomy^201^. Histological samples were taken from the major organs according to a standard protocol. Lung sampling was performed from three independent parts of the potentially infiltrated lung parenchyma.

### Ethical statement

The study protocol was approved by the Ethics Committee of the University Hospitals of Debrecen, Hungary (MK-JA/50/0096-01/2017) and carried out in accordance with the approved guidelines. Informed consent was obtained from the participants in the study.

## Supplementary Information

Supplementary Figure 1.

Supplementary Figure 2.

Supplementary Figure 3.

Supplementary Figure 4.

## Data Availability

All sequence data used in the analyses were deposited in the Sequence read Archive (SRA) (http://www.ncbi.nlm.nih.gov/sra) under PRJNA754268 accession number.
