# Supplementary material for: Circulating microRNA sequencing revealed miRNome patterns in hematology and oncology patients aiding the prognosis of invasive aspergillosis
Source: Sci Rep. 2022 May 3;12:7144. doi: 10.1038/s41598-022-11239-z (PMC9065123; doi:10.1038/s41598-022-11239-z)
Supplement: Supplementary file 1 — Supplementary Figure 1. [file 41598_2022_11239_MOESM1_ESM.docx]

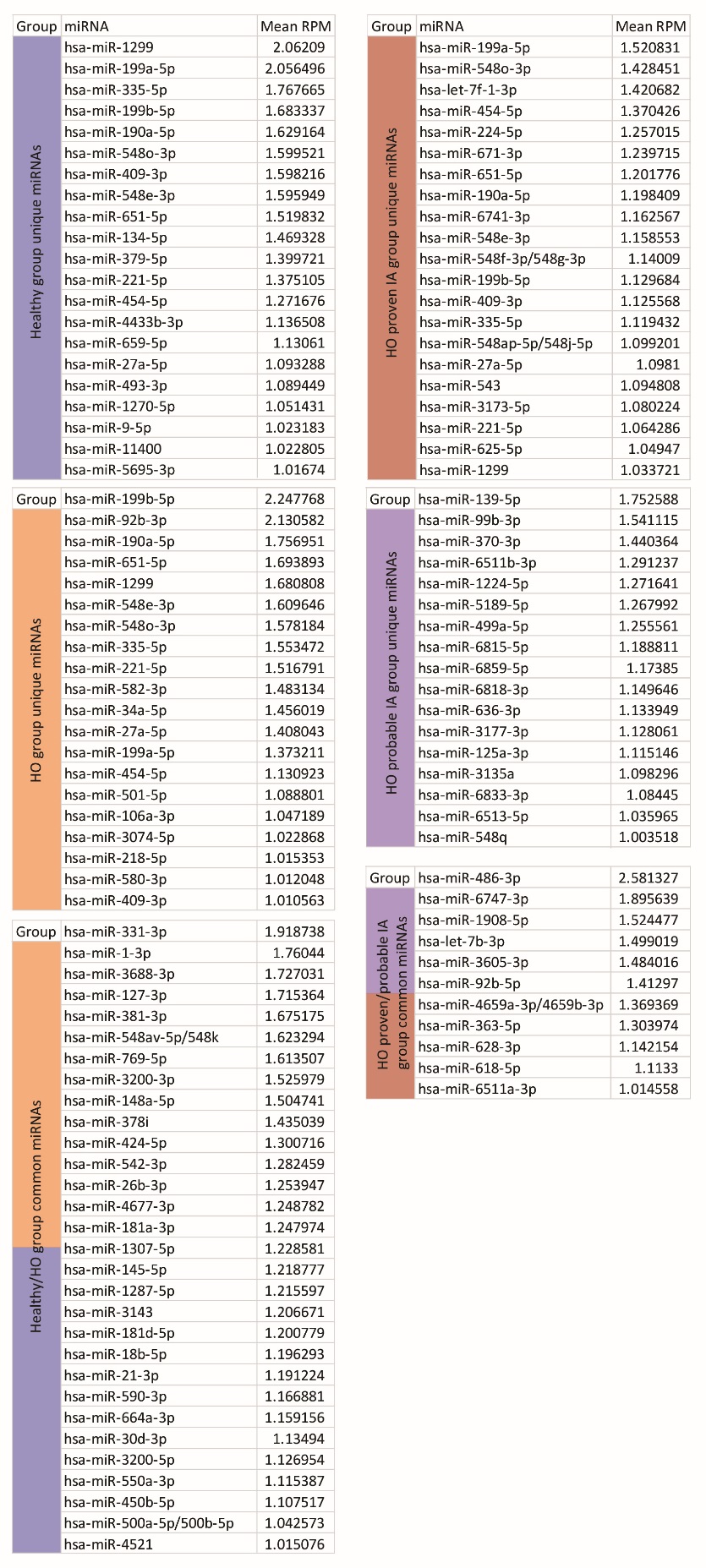


**Supplementary Fig. 1.** **List of patient group-specific miRNAs.** According to the Venn-diagram (Fig. 1) several experimental group-specific miRNAs were identified. Among these neither showed remarkable abundance (RPM<10) thus the source of difference may be the technical variance of sequencing, for this reason these miRNAs were omitted from further analysis.
