# Supplementary material for: Circulating microRNA sequencing revealed miRNome patterns in hematology and oncology patients aiding the prognosis of invasive aspergillosis
Source: Sci Rep. 2022 May 3;12:7144. doi: 10.1038/s41598-022-11239-z (PMC9065123; doi:10.1038/s41598-022-11239-z)
Supplement: Supplementary file 2 — Supplementary Figure 2. [file 41598_2022_11239_MOESM2_ESM.docx]

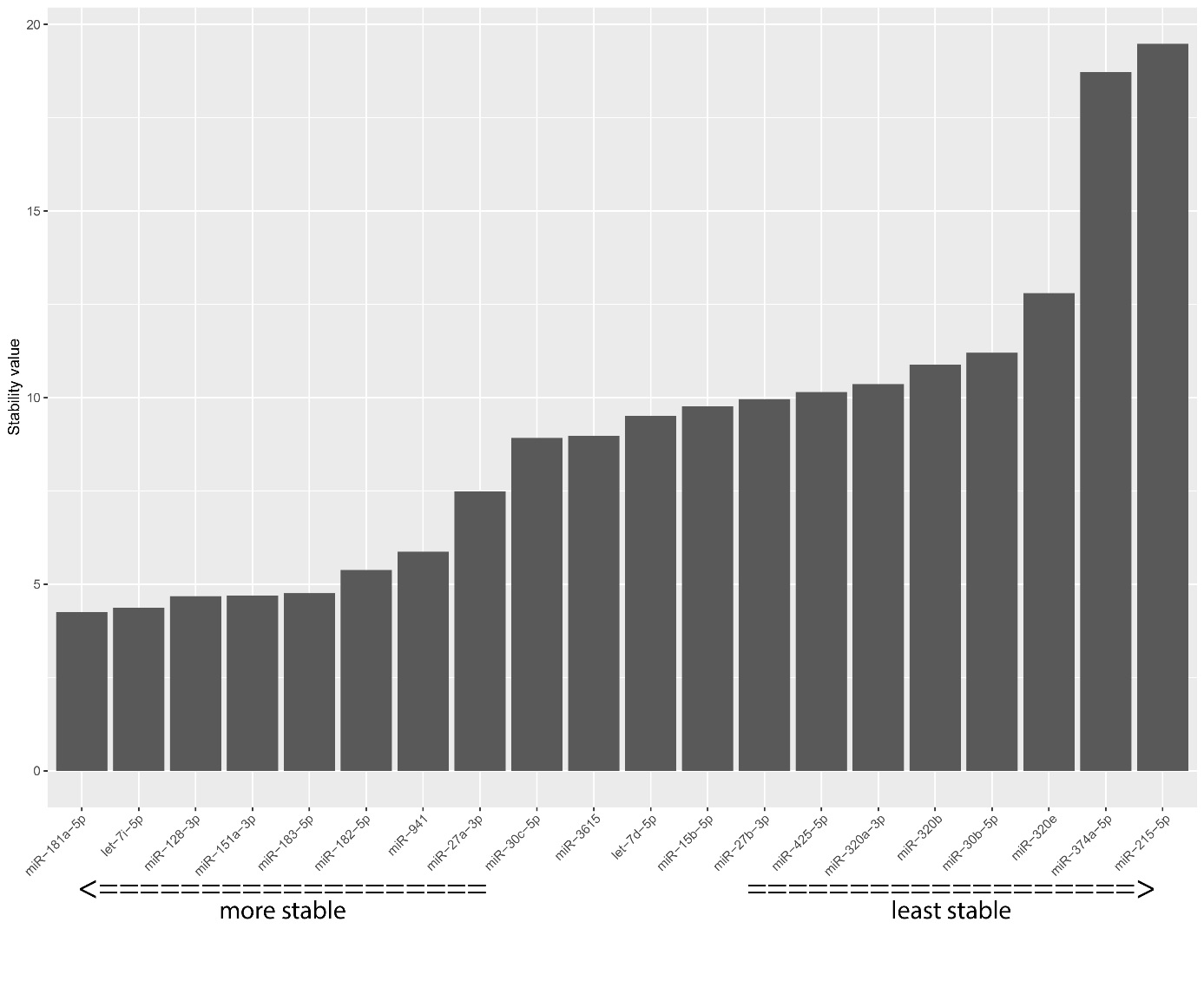


**Supplementary Fig. 2.** **Reffinder results of the potential qRT-PCR normalizer miRNAs.** Based on the sequencing data 20 miRNAs with high abundance showed no significant difference between the groups. The selected miRNAs were analyzed with the Reffinder software and based on the stability value the hsa-miR-181a-5p was used as endogenous control in the qRT-PCR measurements.
