# Supplementary material for: Circulating microRNA sequencing revealed miRNome patterns in hematology and oncology patients aiding the prognosis of invasive aspergillosis
Source: Sci Rep. 2022 May 3;12:7144. doi: 10.1038/s41598-022-11239-z (PMC9065123; doi:10.1038/s41598-022-11239-z)
Supplement: Supplementary file 3 — Supplementary Figure 3. [file 41598_2022_11239_MOESM3_ESM.docx]

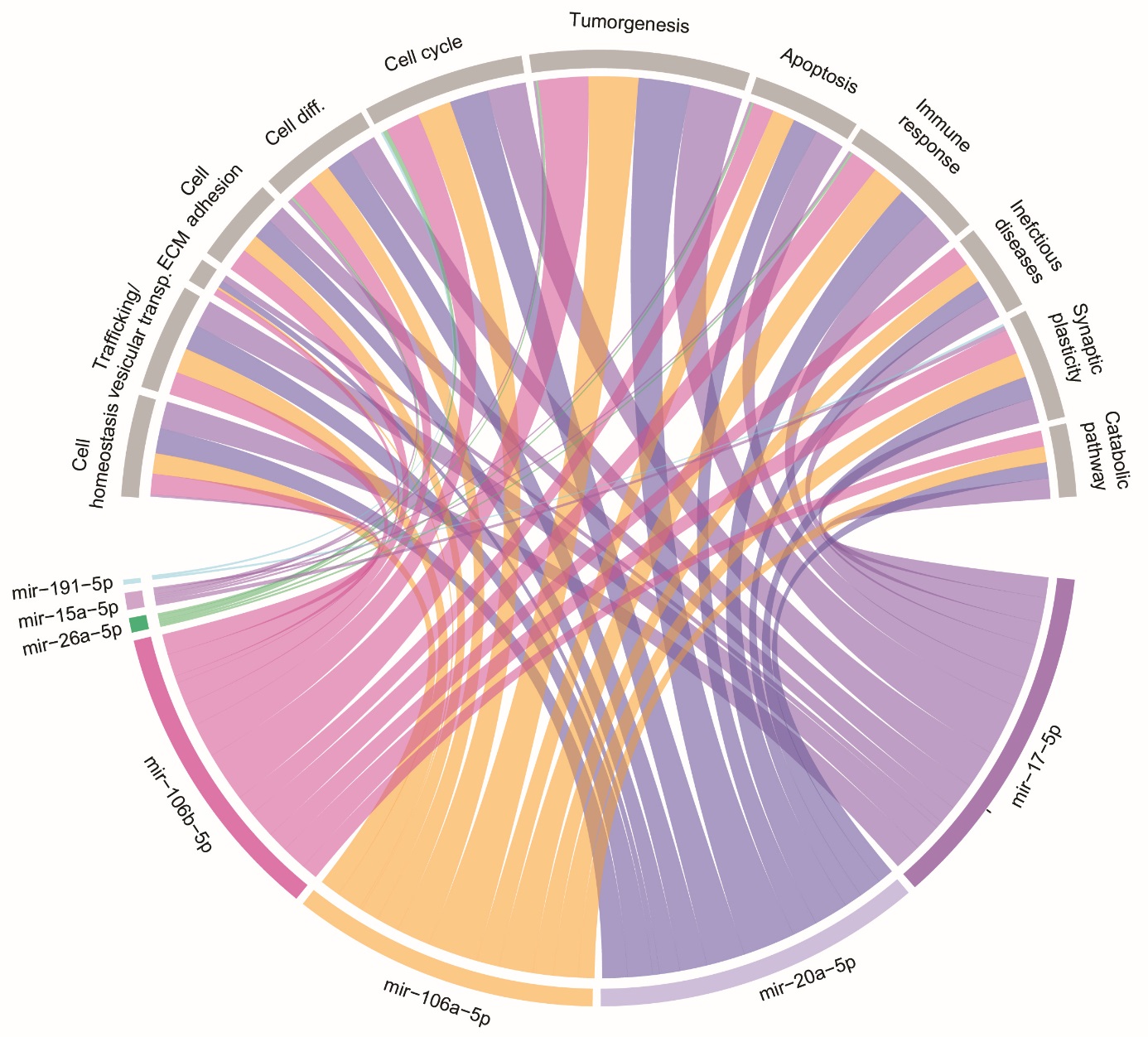


**Supplementary Fig. 3. Interaction network of IA related dysregulated miRNAs with their target genes.** Information gathered from the KEGG database revealed 12 possible biological pathways that can be connected to the selected miRNAs.
