# Supplementary material for: Circulating microRNA sequencing revealed miRNome patterns in hematology and oncology patients aiding the prognosis of invasive aspergillosis
Source: Sci Rep. 2022 May 3;12:7144. doi: 10.1038/s41598-022-11239-z (PMC9065123; doi:10.1038/s41598-022-11239-z)
Supplement: Supplementary file 4 — Supplementary Figure 4. [file 41598_2022_11239_MOESM4_ESM.docx]

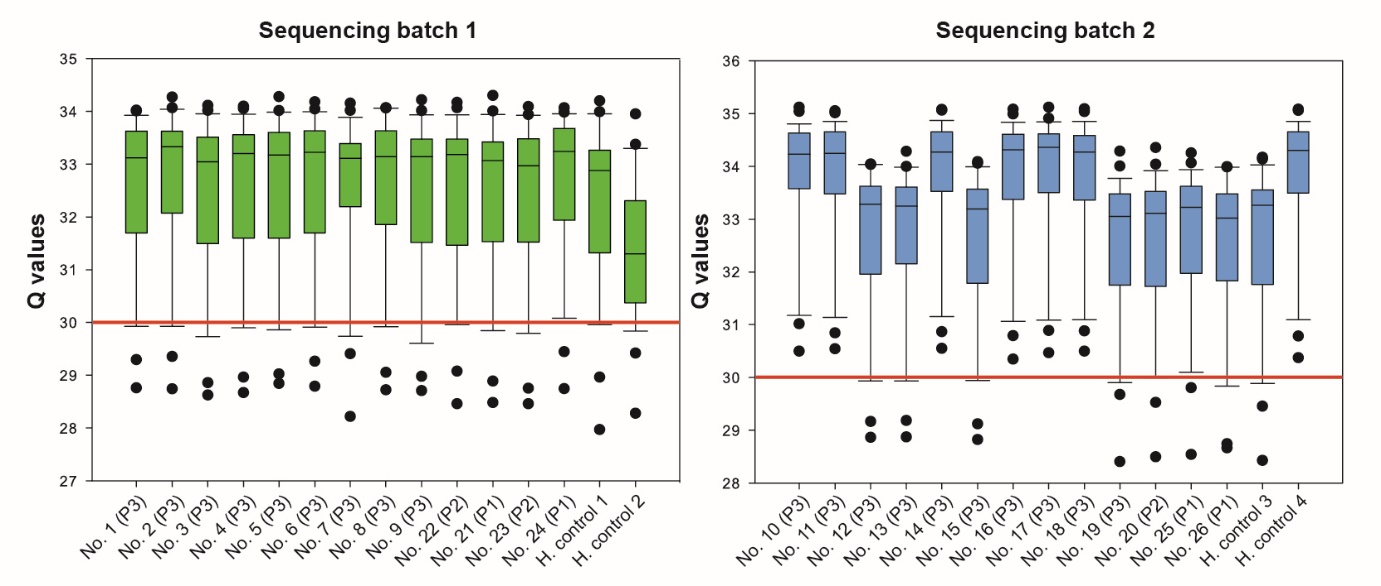


**Supplementary Fig. 4. A summary of sequencing quality scores per bases according to FastQC report.** Quality scores per samples were visualized with boxplots according to the two sequencing runs (batches). A straight red line indicates the Q30 value.
